# Protective mechanism of action of the antifungal drug naftifine against *Mycobacterium abscessus* infection

**DOI:** 10.1128/aac.01105-25

**Published:** 2026-01-14

**Authors:** Jia Wang, Ruchi Paroha, Jian Sha, Atul K. Verma, Blake H. Neil, Paul B. Kilgore, Emily K. Hendrix, Barbara Brown-Elliott, Ashok K. Chopra, Sunhee Lee

**Affiliations:** 1 Department of Microbiology and Immunology, University of Texas Medical Branch 547647, Galveston, Texas, USA; 2 Mycobacteria/Nocardia Laboratory, The University of Texas Health Science Center at Tyler, The University of Texas at Tyler School of Medicine 675071, Tyler, Texas, USA; University of Iowa, Iowa City, Iowa, USA

**Keywords:** *Mycobacterium abscessus*, naftifine, FDA-approved drugs, MmpL3

## Abstract

*Mycobacterium abscessus,* a rapidly growing nontuberculous mycobacterium, causes chronic pulmonary infections that are difficult to treat due to extensive intrinsic drug resistance. Through high-content screening of 786 FDA-approved drugs against intracellular *M. abscessus* in human THP-1 macrophages, we identified naftifine, an antifungal allylamine, as a novel antimycobacterial agent with dual-acting therapeutic mechanisms. Naftifine demonstrated potent activity against reference strains and multidrug-resistant clinical isolates. It showed enhanced efficacy in intracellular environments compared to axenic culture, indicating significant host-directed effects. Mechanistic investigations revealed that naftifine operates through a unique dual mechanism. It directly targets bacteria by inhibiting MmpL3 (MAB_4508), the essential mycolic acid transporter, and modulates host immunity through autophagy activation via the mTOR pathway suppression. Whole-genome sequencing of spontaneous naftifine-resistant mutants identified point mutations (S302T and V299G) in MmpL3. Complementation studies confirmed MmpL3 as the primary molecular target. Cross-resistance analysis with other MmpL3 inhibitors (BM212 and AU1235) validated this target identification. Notably, naftifine represents the first MmpL3 inhibitor demonstrated to induce autophagy, distinguishing it from other MmpL3-targeting compounds. Naftifine-induced autophagy enhanced macrophage-mediated bacterial clearance and reduced infection-associated necrosis, improving host cell survival. *In vivo* studies demonstrated a significant reduction of pulmonary and splenic bacterial burden with reduced lung inflammation. Furthermore, naftifine exhibited synergistic activity with β-lactam antibiotics without antagonizing other clinically used antibiotics. This is the first report demonstrating the unique combination of MmpL3 inhibition and autophagy induction by a single compound against *M. abscessus*, establishing naftifine as a promising dual-action therapeutic candidate for treating multidrug-resistant infections.

## INTRODUCTION

*Mycobacterium abscessus* is the most clinically significant, rapidly growing nontuberculous mycobacterium (NTM), causing pulmonary disease in cystic fibrosis (CF) patients worldwide. The organism’s estimated prevalence rate is 13% in Europe and 16% in the United States among CF patients (1). In addition to infecting patients with a history of pulmonary disorders such as CF, bronchiectasis, and chronic obstructive pulmonary disease, *M. abscessus* can also infect otherwise healthy individuals with no apparent risk factors (2). It is notably the most challenging NTM infection to treat, with a dismal treatment success rate of only 45.6% (2).

This poor outcome is attributed mainly to the fact that *M. abscessus* is notoriously resistant to antibiotics. Current treatment guidelines for CF patients recommend an 18-month multidrug regimen, beginning with an intensive phase lasting 3–12 weeks, followed by a continuation phase (3). The intensive phase typically includes two intravenous antibiotics, such as amikacin, imipenem, tigecycline, or cefoxitin, combined with one or more oral drugs, including macrolides (clarithromycin and azithromycin), clofazimine, linezolid, and occasionally fluoroquinolones like ciprofloxacin or moxifloxacin (3). Two or more oral antibiotics are prescribed for the continuation phase, usually including a macrolide and inhaled antibiotics, such as amikacin (3, 4). For non-CF patients, the 2020 ATS/IDSA (American Thoracic Society/Infectious Diseases Society of America) guidelines recommend a minimum of three active antibiotics based on *in vitro* susceptibility testing (5). However, the optimal drug combinations and treatment duration remain undefined, and no current antibiotic regimen reliably cures *M. abscessus* lung infections. Surgical resection combined with chemotherapy remains the only consistently curative option for localized disease in non-CF patients (6). This underscores the urgent need to identify new therapeutic agents.

Unfortunately, previous high-throughput drug screens targeting *M. abscessus* have yielded few effective candidates (7–14). Most drug discovery efforts for *M. abscessus* focus on either repurposing existing antibiotics or evaluating tuberculosis (TB) drugs for cross-activity (15). Notably, when the same compound library was screened against *M. tuberculosis,* TB-causing mycobacteria, and *M. abscessus*, the hit rate for *M. abscessus* was approximately 10-fold lower (16). Unlike *M. tuberculosis, M. abscessus* thrives in environmental reservoirs like soil and water and has developed evolutionary adaptations to survive in hostile environments. These include drug-modifying enzymes, reduced membrane permeability due to a lipid-rich cell wall, and extensive drug efflux systems such as ATP-binding cassette transporters and mycobacterial membrane large proteins (MmpL), which limit antibiotic accumulation within the cell (17). Resistance to macrolides, the primary drug recommended for treating *M. abscessus* lung diseases, is often mediated by the *erm*(41) gene, which encodes a ribosomal methylase that modifies the 23S rRNA to confer inducible resistance (18). Additionally, *M. abscessus* displays smooth or rough colony morphologies based on the expression of surface glycopeptidolipids, which influence both virulence and antibiotic susceptibility (19).

In this study, we screened a library of 786 FDA-approved drugs to identify compounds that inhibit the intracellular growth of *M. abscessus* in human THP-1 macrophages. From this screen, 21 potential candidates were identified, including six non-antibacterial agents selected for further evaluation. Among them, naftifine, an antifungal allylamine, emerged as the most potent at reducing intracellular bacterial burden, significantly inhibiting *M. abscessus* growth without inducing cytotoxic effects in host macrophages. Naftifine exhibited robust anti-*M*. *abscessus* activity in *in vitro* macrophage and *in vivo* immunosuppressed mouse infection models. Mechanistic investigations revealed that naftifine acts through a dual mode of action: directly inhibiting bacterial growth and enhancing host-cell defense mechanisms. Naftifine reduced macrophage necrosis and activated autophagy by inhibiting the mTOR (mammalian target of rapamycin) signaling pathway. Furthermore, whole-genome sequencing of spontaneous naftifine-resistant mutants revealed mutations in *MAB_4508*, which encodes the MmpL3 protein, an essential inner membrane transporter responsible for exporting mycolic acid precursors, the key components of the mycobacterial outer membrane. These findings strongly implicate MmpL3 as the likely molecular target of naftifine. To our knowledge, this is the first report demonstrating the anti-*M*. *abscessus* effects of naftifine, highlighting its promise as a therapeutic candidate for adjunctive treatment of *M. abscessus* infections.

## RESULTS

### Identification of FDA-approved drugs that inhibit the intracellular growth of *M. abscessus*

To identify drugs with potential activity against *M. abscessus*, we screened a library of 786 compounds using the Opera Phenix high-content imaging system. The objective was to identify compounds that selectively inhibit intracellular *M. abscessus* replication within THP-1 macrophages while exhibiting minimal or no cytotoxic effects on host cells. For this purpose, we engineered the *M. abscessus* ATCC 19977 type strain (ATCC 19977^T^) to express the red fluorescent protein tdTomato, enabling quantification of intracellular bacterial burden via fluorescence intensity. Drug treatment was initiated 4 hours after infection to allow bacterial uptake and establishment within macrophages. The impact of the compound library on intracellular bacterial growth was then evaluated 48 hours post-infection. Each screening plate included bedaquiline (1.5 μM) as a positive control and 0.1% dimethyl sulfoxide (DMSO) as a vehicle control. Bedaquiline is an FDA-approved drug that treats drug-resistant TB and shows potent activity against *M. abscessus in vitro* (20). It has also been used as a reference compound in previous drug screens for *M. abscessus* (11, 12). The assay was performed in five independent screening runs, yielding a Z′ factor of 0.71, indicating high assay quality and statistical robustness. Compounds were classified as hits if they inhibited intracellular *M. abscessus*-tdTomato fluorescence by more than 25%, while maintaining at least 60% viability of THP-1 cells, relative to the DMSO-treated control on each plate.

**Fig 1:**
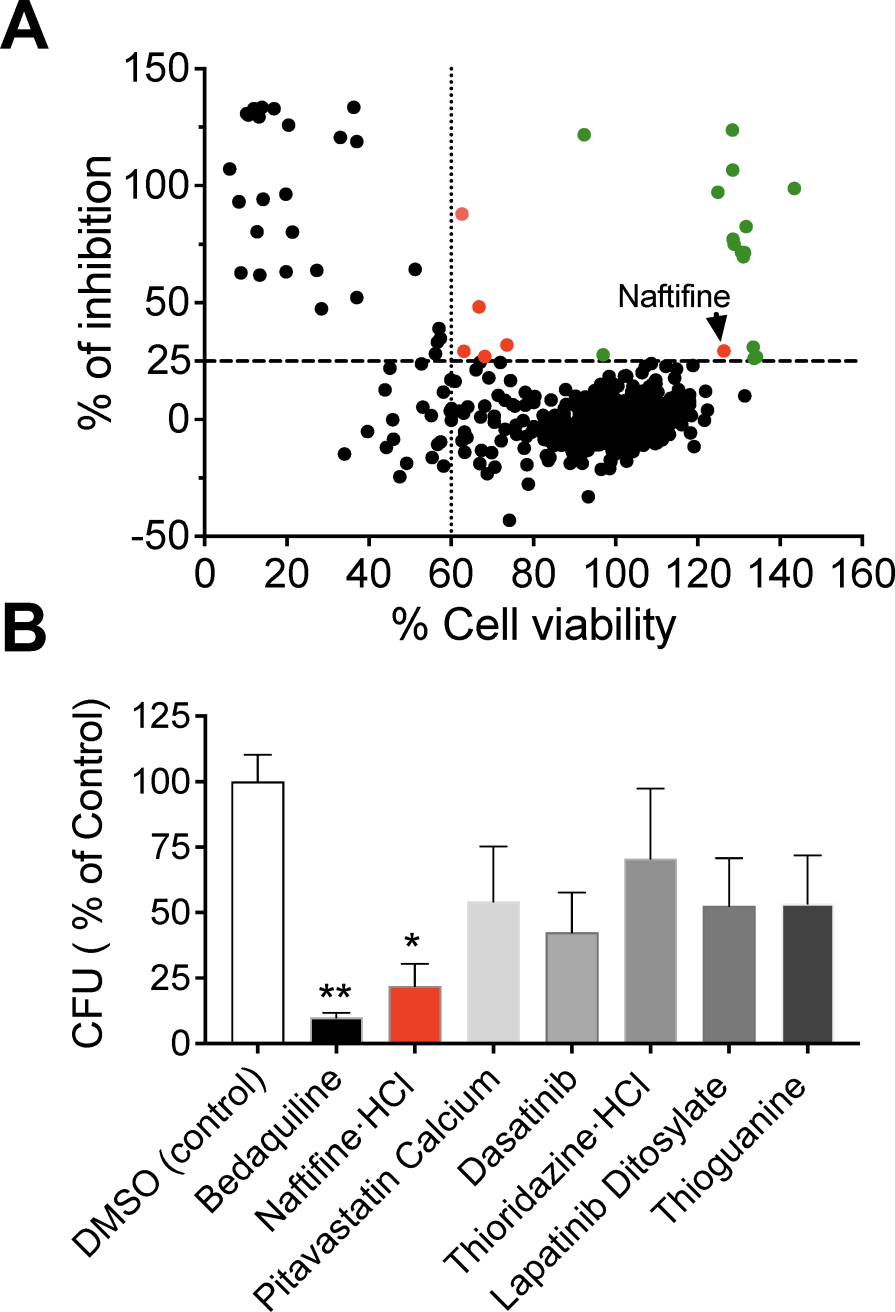
High-content screening identifies naftifine as an anti-mycobacterial compound. (**A**) A high-content screen was performed using an FDA-approved drug library in *M. abscessus*-infected THP-1 macrophages at 48 hours post-infection. Compounds were tested at 10 µM and evaluated for their ability to inhibit intracellular bacterial growth (*y*-axis) while maintaining host cell viability (*x*-axis). The horizontal dotted line represents the threshold for bacterial inhibition (25%), and the vertical dotted line indicates the minimum acceptable cell viability (60%). Black dots represent individual test compounds, green dots indicate known antibiotics, and red dots highlight non-antibacterial compounds that met the hit criteria. The position of naftifine is indicated with an arrow. (**B**) Selected non-antibacterial hits were validated by CFU enumeration assays. THP-1 cells were infected with *M. abscessus* and treated with 10 μM of the six top-scoring compounds for 48 hours. The intracellular bacterial burden was quantified by CFU enumeration and expressed as a percentage of the DMSO control. Bedaquiline at 1.5 μM served as a positive control antibiotic. Data represent the mean ± SD. Statistical significance was determined by one-way ANOVA followed by Dunnett’s multiple comparison test for comparisons against the DMSO control. *P < 0.05; **P < 0.01.

In total, 21 candidate compounds met these criteria and appeared in the top-right quadrant of the inhibition-viability plot (Fig. 1A). Among these, 15 were classified as antibiotics (highlighted as green dots). Rifabutin, bleomycin sulfate, and clarithromycin were noteworthy among them, each showing over 100% inhibition, equivalent to or exceeding the effect of bedaquiline. Rifabutin, supported by prior *in vitro* and *in vivo* studies, has shown promise in treating *M. abscessus* and may benefit patients with drug-refractory infections (21, 22). Bleomycin sulfate, a glycopeptide antibiotic produced by *Streptomyces verticillus* and primarily used in chemotherapy for its antineoplastic properties, exhibited notable *in vitro* activity against *M. abscessus* with relatively low cytotoxicity in our screening assays. However, its well-established risk of dose-limiting pulmonary toxicity significantly constrains its potential for repurposing as an antimicrobial agent in this context (23). Clarithromycin, a standard component of current treatment regimens for *M. abscessus*, also demonstrated strong activity (18). Several other known antibiotic classes were also validated by the screen, including macrolides (erythromycin and azithromycin) (24), oxazolidinone (linezolid) (25), and fluoroquinolones (gatifloxacin, ciprofloxacin, moxifloxacin, and levofloxacin) (26–28). Additional hits included telithromycin (a ketolide), lincosamides (clindamycin and lincomycin), the aminoglycoside capreomycin, and the tetracycline demeclocycline (28–30).

To evaluate the activity of non-antibacterial agents identified in the screen, we selected the remaining six candidates (highlighted as red dots) for follow-up testing and quantified intracellular bacterial survival using colony-forming unit (CFU) enumeration. These included pitavastatin calcium (a lipid-lowering statin), naftifine HCl (an antifungal), thioridazine HCl (an antipsychotic), and three antineoplastic agents (dasatinib, lapatinib ditosylate, and thioguanine). Among them, treatment with naftifine HCl showed the most pronounced reduction in intracellular *M. abscessus* burden (Fig. 1B). Naftifine HCl is an allylamine derivative commonly used for treating topical fungal infections by inhibiting squalene epoxidase, leading to toxic accumulation of squalene and fungal cell death (31). Since naftifine’s activity against *M. abscessus* has not been previously documented, and *M. tuberculosis* lacks the genes coding for squalene epoxidase (32), we selected naftifine for further evaluation.

### Naftifine inhibits the intracellular growth of *M. abscessus*

**Fig 2:**
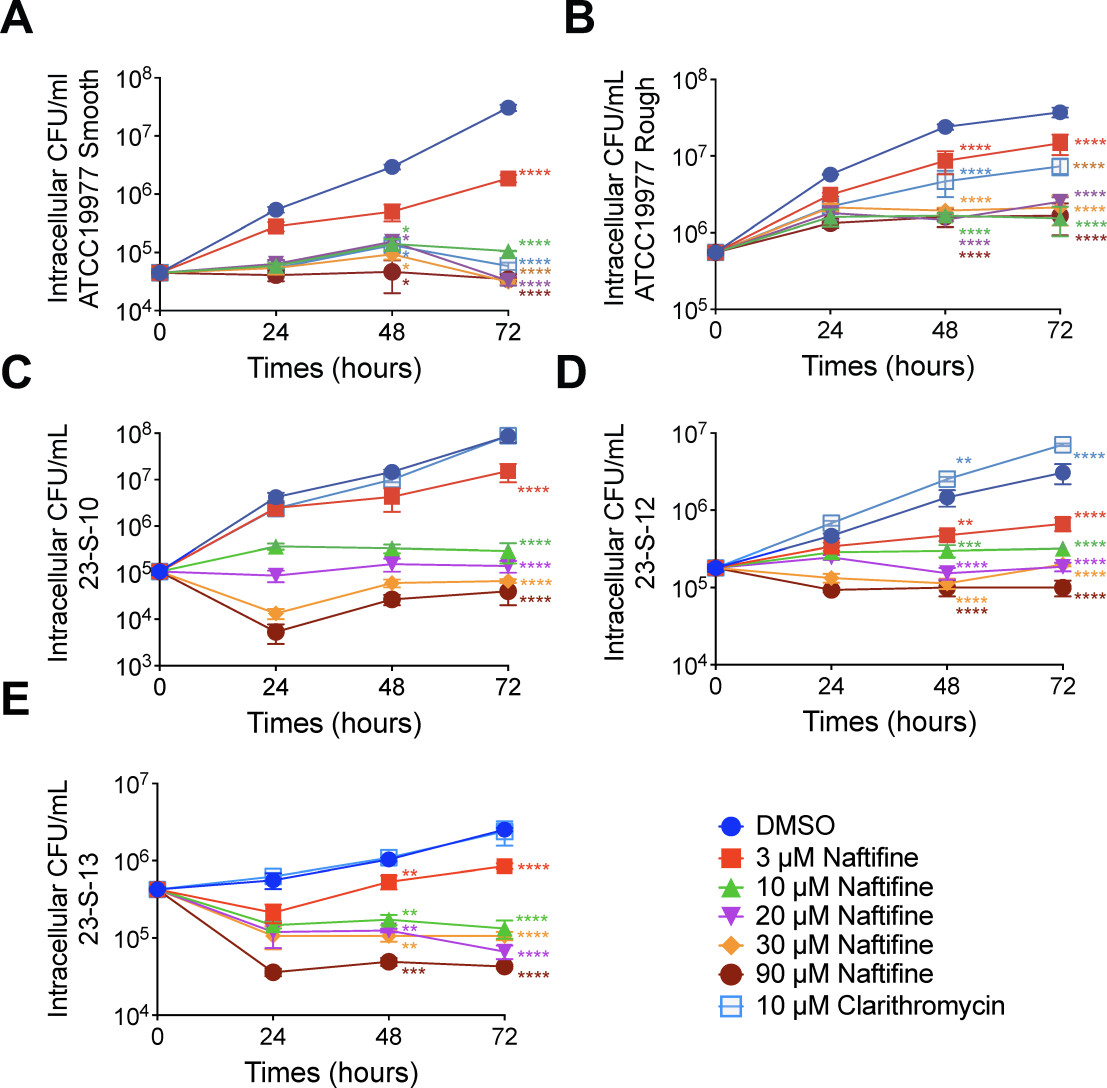
Naftifine inhibits intracellular *M. abscessus* growth in a concentration- and time-dependent manner. THP-1 macrophages were infected with (**A**) ATCC 19977^T^ smooth variant, (**B**) ATCC 19977^T^ rough variant, and clinical isolates (**C**) 23-S-10, (**D**) 23-S-12, and (**E**) 23-S-13. The ATCC 19977^T^ strains are sensitive to both clarithromycin and amikacin, while the clinical isolates are resistant to both antibiotics. Following infection, cells were treated with increasing concentrations of naftifine (3–90 μM) or 10 μM clarithromycin as a control. Intracellular bacterial burden was quantified by CFU enumeration at 24, 48, and 72 hours post-treatment. Data represent mean ± SD from three independent experiments. Statistical significance was determined by two-way ANOVA with Tukey’s multiple comparisons test for comparisons between each naftifine concentration and the DMSO control at corresponding time points. **P < 0.01, ***P < 0.001, and ****P < 0.0001.

To assess the efficacy of naftifine against intracellular *M. abscessus*, we tested a range of bacterial strains, treatment durations, and drug concentrations in THP-1 macrophages. The tested strains included the reference ATCC 19977^T^ strain in both smooth and rough morphotypes and clinical isolates of *M. abscessus* subsp. *abscessus* harboring mutations that confer resistance to amikacin and clarithromycin (Table 1). Naftifine was evaluated post-infection at concentrations of 3, 10, 20, 30, and 90 µM. Intracellular bacterial burden was measured at 24, 48, and 72 hours post-infection. Clarithromycin (10 µM) was used as a control (Fig. 2).

**TABLE 1:** MIC of naftifine against *M. abscessus* subsp*. abscessus* strains

Strain	Morphotype	Mutational resistance		MIC (μg/mL) of naftifine
		Clarithromycin	Amikacin	
ATCC 19977^T^	Smooth	−	−	16
ATCC 19977^T^	Rough	−	−	16
23-S-10 (clinical isolate 595)	Smooth	+	+	16
23-S-12 (clinical isolate 612)	Smooth	+	+	16
23-S-13 (clinical isolate 649)	Smooth	+	+	16

In THP-1 macrophages infected with the ATCC 19977^T^ smooth strain, treatment with naftifine at 10 µM resulted in a significant reduction of intracellular bacterial load by 1.3 log_10_ CFU at 48 hours post-infection, compared to the untreated (DMSO) control (Fig. 2A). By 72 hours, even the lowest tested concentration (3 µM) achieved a significant reduction of 1.2 log_10_ CFU, while 10 µM led to a more substantial reduction of 2.5 log_10_ CFU compared to vehicle control at 72 hours post-infection (Fig. 2A). Similar inhibitory effects were observed in the ATCC 19977^T^ rough morphotype and all tested drug-resistant clinical isolates. Naftifine, even at 3 µM, significantly reduced intracellular bacterial loads at either 48 or 72 hours post-infection (Fig. 2B through E). At 72 hours post-infection, treatment with 10 µM naftifine led to consistent reductions in CFU counts: 1.5 log_10_ for ATCC 19977^T^ rough strain, 2.5 log_10_ for clinical isolate 23-S-10, 0.9 log_10_ for 23-S-12, and 1.3 log_10_ for 23-S-13 (Fig. 2B through E). In contrast, clarithromycin failed to inhibit the intracellular growth in all drug-resistant clinical isolates. Notably, the 10 µM naftifine treatment consistently produced substantial bacterial reductions, comparable to those at higher concentrations, highlighting its effectiveness even at low doses.

### Host cell viability is maintained during naftifine treatment of *M. abscessus*-infected macrophages

**Fig 3:**
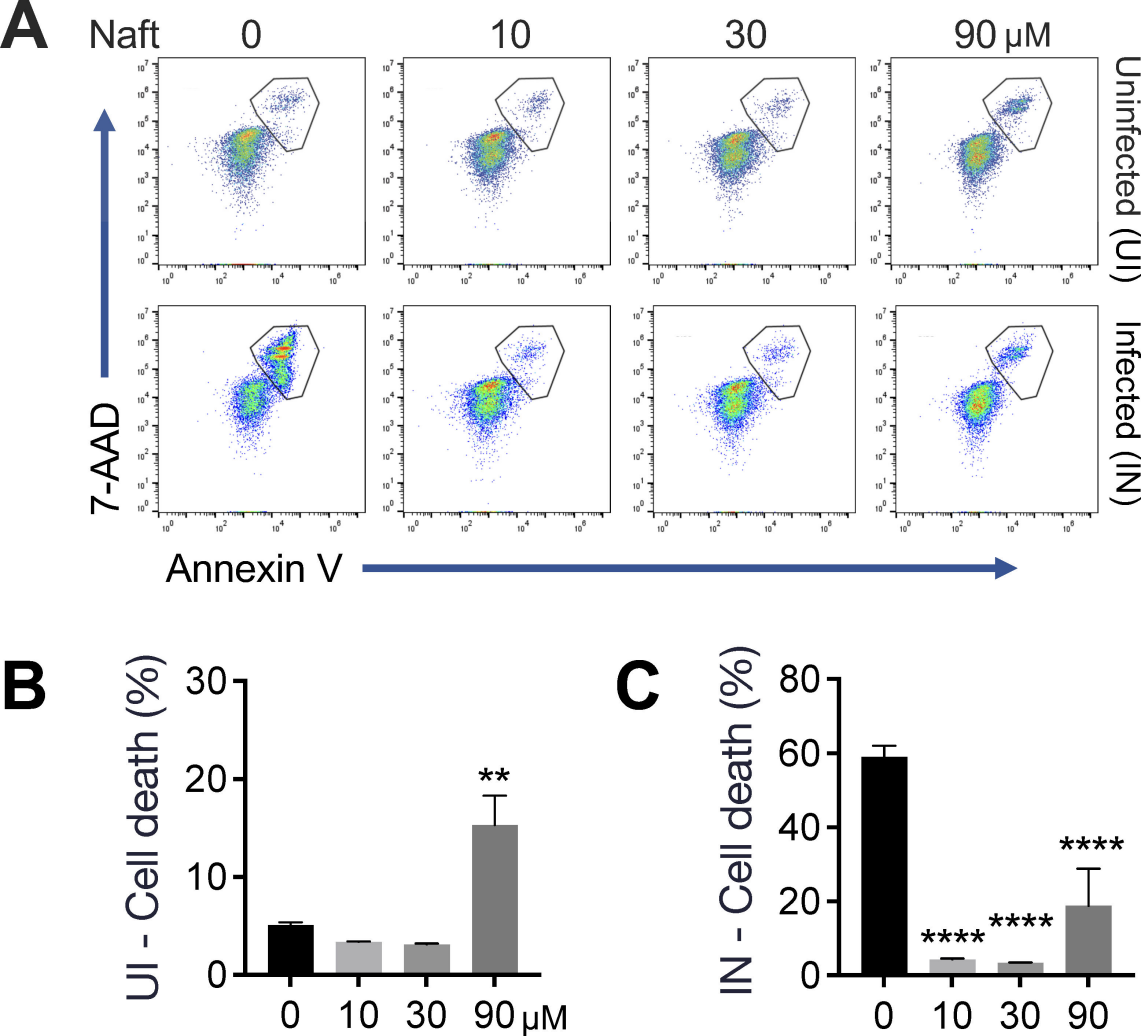
Dose-dependent cytotoxicity analysis in uninfected and *M. abscessus*-infected cells. (**A**) Representative flow cytometry dot plots show Annexin V and 7-AAD staining patterns in uninfected (UI) and infected (IN) cells treated with increasing concentrations (0, 10, 30, and 90 μM) of the test compound. THP-1 cells were infected with *M. abscessus* ATCC 19977^T^ smooth variants and treated with naftifine for 3 days. Gated regions indicate dead cells (Annexin V^+^, including early and late apoptotic and necrotic populations). (**B and C**) Quantification of cell death shows the percentage of Annexin V^+^ population (both 7-AAD^+^ and 7-AAD⁻) in uninfected (**B**) and infected (**C**) cells. Data represent mean ± SEM from at least three independent experiments. Statistical significance was determined by one-way ANOVA followed by Dunnett’s *post hoc* test for multiple comparisons against the control. **P < 0.01 and ****P < 0.0001 compared to untreated control (0 μM).

To determine whether the observed reduction in intracellular bacterial burden was due to direct naftifine-induced cytotoxicity, we evaluated the viability of uninfected and *M. abscessus* ATCC 19977^T^-infected THP-1 cells after 72 hours of naftifine treatment, using an Annexin V and 7-AAD dual staining (Fig. 3A). Annexin V detects phosphatidylserine externalization characteristic of early apoptosis, while 7-AAD, a membrane-impermeable DNA dye, identifies cells with compromised membrane integrity indicative of late apoptosis or necrosis. Viable cells were defined as Annexin V⁻/7-AAD⁻, while early apoptotic cells (Annexin V^+^/7-AAD⁻) and late apoptotic/necrotic cells (Annexin V^+^/7-AAD^+^) were identified by gating as shown in Fig. 3A. Cell death was quantified in both uninfected (Fig. 3B) and infected (Fig. 3C) conditions.

In uninfected macrophages, naftifine at 10 and 30 µM did not significantly affect cell viability, which remained comparable to that of untreated controls. However, treatment with 90 µM naftifine induced significant cytotoxicity, increasing cell death to 15.3% compared to 5.1% in controls (*P* < 0.01), indicating mild toxicity at the highest concentration tested (Fig. 3B).

Infected macrophages displayed a markedly different response profile. Baseline infection with *M. abscessus* substantially compromised cell viability, reflecting significant infection-induced cytotoxicity. Treatment with naftifine improved viability in a clear dose-dependent manner. Cell death decreased from 59.1% in untreated infected cells to 18.8% at 90 µM concentration (Fig. 3C). This protective effect is likely due to naftifine’s antimicrobial activity, reducing bacterial burden and subsequent infection-mediated host cell damage. These findings suggest that naftifine not only effectively limits intracellular *M. abscessus* replication but may also preserve host cell viability, demonstrating therapeutic potential with minimal direct cytotoxicity.

### Naftifine directly inhibits the growth of *M. abscessus*

**Fig 4:**
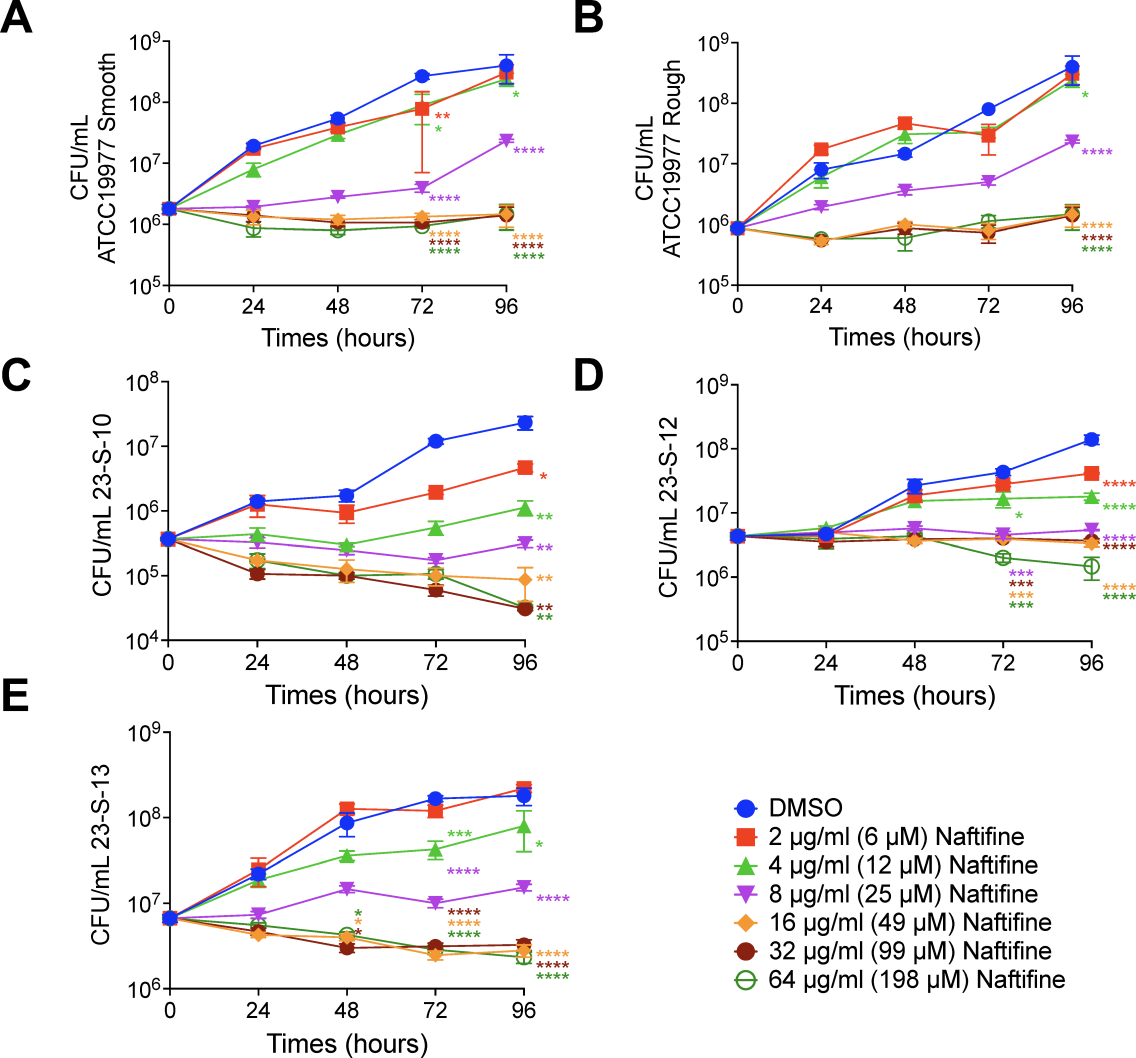
Naftifine exhibits bacteriostatic activity against *M. abscessus* in axenic culture. Time-kill assays were performed to evaluate the antimicrobial activity of naftifine against different *M. abscessus* strains and morphological variants. (**A**) ATCC 19977^T^ smooth variant, (**B**) ATCC 19977^T^ rough variant, and clinical isolates (**C**) 23-S-10, (**D**) 23-S-12, and (**E**) 23-S-13. The ATCC 19977^T^ strains are sensitive to both clarithromycin and amikacin, while the clinical isolates are resistant to both antibiotics. Bacterial cultures in logarithmic growth phase were treated with naftifine at concentrations ranging from 2 to 64 µg/mL (equivalent to 6–198 μM) or DMSO vehicle control. Samples were collected at 24-hour intervals over a 96-hour incubation period, and bacterial viability was assessed by CFU enumeration following serial dilution plating. Data represent mean ± SEM from two independent experiments performed in triplicate. Statistical comparisons between naftifine-treated and DMSO control at each time point were performed using two-way ANOVA with Tukey’s multiple comparisons test. Statistical significance is indicated as *P < 0.05, **P < 0.01, ***P < 0.001, and ****P < 0.0001.

After confirming naftifine’s intracellular activity against various *M. abscessus* strains, we evaluated its direct antibacterial effect in axenic culture. Naftifine was evaluated at concentrations ranging from 0.25 to 128 µg/mL in twofold increments. As shown in Table 1, both reference and clinical strains exhibited a minimum inhibitory concentration (MIC) of 16 µg/mL (49 µM). To further assess the drug’s potency and kinetics, we performed 4-day *in vitro* time-kill assays using the same panel of strains. Naftifine was tested at concentrations from 2 to 64 µg/mL (6–198 μM). All *M. abscessus* strains showed significant growth inhibition at naftifine concentrations as low as 4 µg/mL (12 μM), with measurable reductions in CFU beginning by day 3 (Fig. 4A through E). However, naftifine’s activity in axenic culture was notably less pronounced than in intracellular settings. On day 3 at 12 μM, CFU reductions in axenic culture were modest: 0.6 and 0.4 log_10_ for the ATCC 19977^T^ smooth and rough morphotypes, and 1.4, 0.5, and 0.6 log_10_ for clinical isolates 23-S-10, 23-S-12, and 23-S-13, respectively (Fig. 4). In contrast, the same concentration of naftifine produced markedly greater reductions in intracellular bacterial load: 2.5, 1.5, 2.5, 0.9, and 1.3 log_10_, respectively (Fig. 2), suggesting enhanced efficacy in the intracellular environment.

As expected, naftifine at 4 µg/mL (12 µM) resulted in moderate reductions in CFU, ranging from 0.4 to 1.4 log_10_ in drug-resistant clinical isolates after 3–4 days of exposure (Fig. 4C through E). Increasing the concentration to 8 µg/mL (25 µM) led to more consistent and substantial reductions of approximately 1.1–1.9 log_10_ across all tested strains (Fig. 4A through E). Notably, raising the dose to 64 µg/mL, four times the MIC, did not improve bacterial inhibition beyond the level achieved at 16 µg/mL, suggesting a plateau in efficacy. To determine whether naftifine acts as a bactericidal or bacteriostatic agent against *M. abscessus*, we compared CFU counts at various times to the baseline count on Day 0. A ≥3 log_10_ (99.9%) reduction in CFUs is the accepted benchmark for bactericidal activity. Since naftifine failed to produce this level of reduction at all tested concentrations, we conclude that it exhibits bacteriostatic activity within this concentration range.

### Naftifine is active against *M. abscessus* in a mouse model

In immunocompetent BALB/c mice, *M. abscessus* infection, whether introduced via aerosolization or intravenous injection, fails to establish a persistent infection, as the bacteria are rapidly cleared from the lungs, spleens, and livers within 4 weeks (33). To overcome this limitation and generate a progressive high level of infection suitable for drug evaluation, we utilized a chemically induced immunosuppressive mouse model. A prior study has established that dexamethasone serves as an effective immunosuppressive agent in C3HeB/FeJ mice infected with *M. abscessus* via aerosol exposure (34). This approach facilitates initial bacterial proliferation and maintains a substantial pulmonary burden of *M. abscessus*, thereby providing a robust framework for assessing the efficacy of antibiotics (34, 35). Similarly, proof-of-concept studies in BALB/c mice have demonstrated that dexamethasone treatment enhances the pulmonary load of *M. abscessus* after bacterial implantation (34), supporting its broader applicability across mouse strains.

We further refined this model by selecting the rough variants, which are frequently isolated from patients suffering from severe, chronic lung infections, notably in individuals with CF, and are linked to a more rapid deterioration in pulmonary function (36–38). Compared to the smooth morphotype, rough variants exhibit increased virulence in both murine and zebrafish models, triggering a stronger inflammatory response and promoting the formation of necrotic granulomas (39–42). Given these clinically relevant characteristics, testing therapeutics against the R morphotype is likely to yield results of greater translational significance.

**Fig 5:**
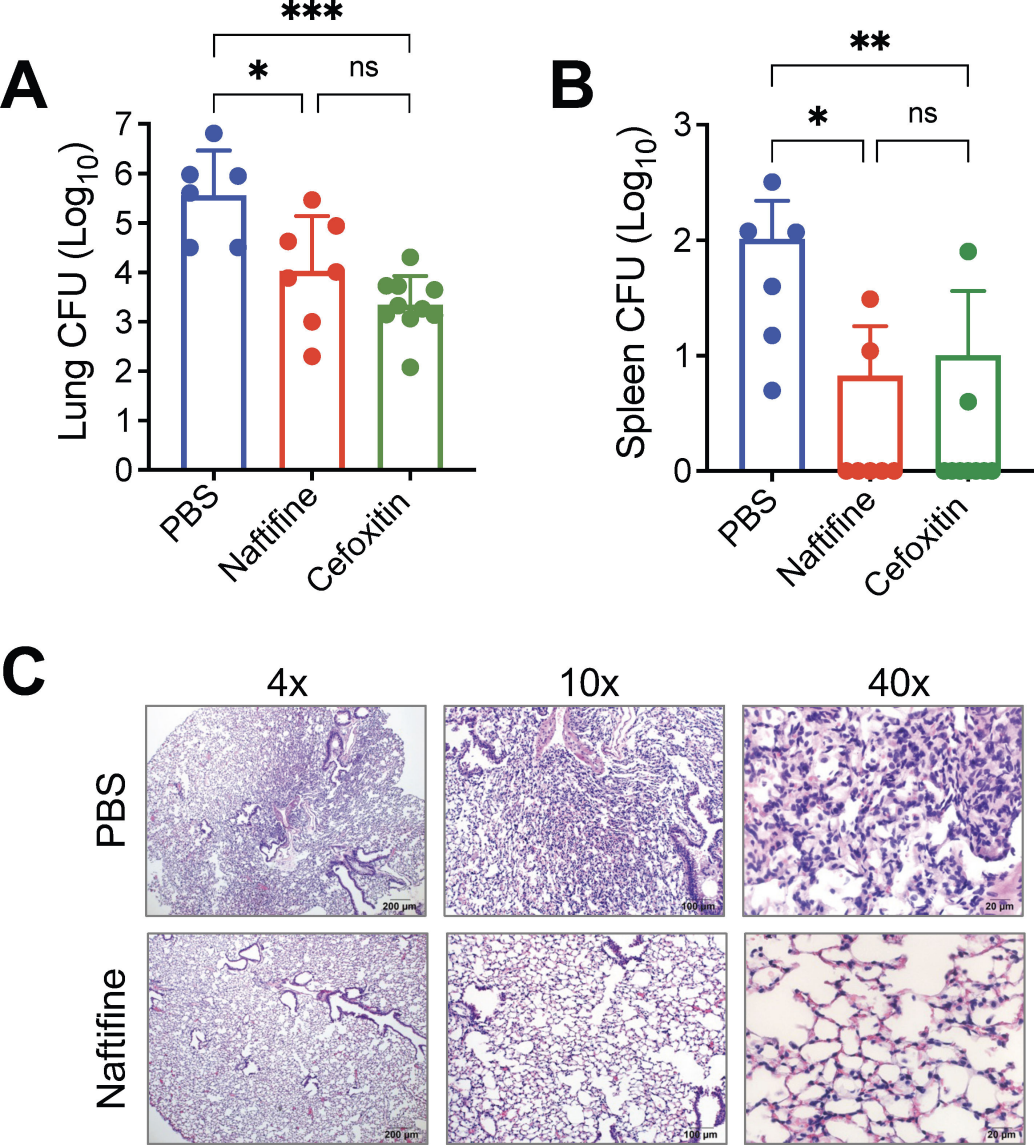
Naftifine reduces *M. abscessus* burden in the lungs and spleens of immunosuppressed mice. BALB/c mice were immunosuppressed with dexamethasone and subsequently infected intranasally with 1,000 CFU of *M. abscessus* ATCC 19977^T^ rough variant. Treatment began 3 days post-infection and continued once daily for 14 days. Mice received either naftifine HCl (50 mg/kg of body weight, intraperitoneal), cefoxitin (200 mg/kg of body weight, subcutaneous, positive control), or PBS vehicle control (intraperitoneal). After the treatment period, mice were euthanized, and bacterial loads in the (**A**) lungs and (**B**) spleens were quantified by CFU enumeration and expressed as log_10_ CFU per organ. Data represent individual mice with mean ± SEM. (**C**) Representative hematoxylin and eosin-stained lung sections from infected mice treated with PBS vehicle control (top panels) or naftifine (bottom panels) are shown at 4×, 10×, and 40× magnifications to illustrate histopathological changes. Statistical comparisons between treatment groups were performed with the Kruskal–Wallis test followed by Dunn’s multiple comparisons test. *P < 0.05; **P < 0.01; *** *P* < 0.001 and ns, not significant.

For our infection model, 6–8-week-old BALB/c mice were immunosuppressed by daily subcutaneous administration of dexamethasone (5 mg/kg of body weight), starting 1 week prior to infection. Mice were then intranasally infected with 1,000 CFU of *M. abscessus* ATCC 19977^T^ rough morphotype, and immunosuppression was maintained for the duration of the study. Naftifine treatment was initiated 3 days post-infection (43) and administered intraperitoneally at 50 mg/kg of body weight/day for 2 weeks. The positive control antibiotic cefoxitin was administered subcutaneously daily at 200 mg/kg of body weight for the same duration. Results demonstrated that both naftifine and the control antibiotic cefoxitin effectively reduced *M. abscessus* burden in infected mice. In the lungs (Fig. 5A), each treatment led to a clear reduction in CFU counts compared to the phosphate-buffered saline (PBS) group, indicating successful suppression of pulmonary infection. A similar trend was observed in the spleen (Fig. 5B), where both treatments lowered bacterial dissemination relative to untreated controls. Reductions were observed consistently across treated animals, with many showing little to no detectable bacteria. These findings support naftifine as a promising antimicrobial candidate with proven efficacy against both pulmonary and systemic *M. abscessus* infection in this murine model, comparable to the performance of the reference antibiotic cefoxitin.

Histopathological examination of lung tissues revealed significant differences between treatment groups. PBS-treated mice showed extensive inflammation characterized by diffuse leukocyte infiltration, alveolar collapse, and parenchymal damage. In contrast, naftifine-treated lungs exhibited a substantial reduction in inflammatory burden and largely preserved tissue structure (Fig. 5C). These findings highlight naftifine’s ability to reduce infection-associated lung pathology by combining antimicrobial activity with host-directed effects. Together with our *in vitro* data, the *in vivo* results further validate its therapeutic potential against *M. abscessus* infection.

### Mutations in MmpL3 confer resistance to naftifine 

To investigate the potential molecular target of naftifine, we isolated two spontaneous naftifine-resistant mutants of *M. abscessus* ATCC 19977^T^ smooth morphotype (designated Naft R1 and Naft R2) and subjected them to whole-genome sequencing. Both mutants exhibited markedly elevated MICs (>1,024 µg/mL or 3.2 mM), in contrast to the parental strain, which had an MIC of 16 μg/mL (49 μM).

**Fig 6:**
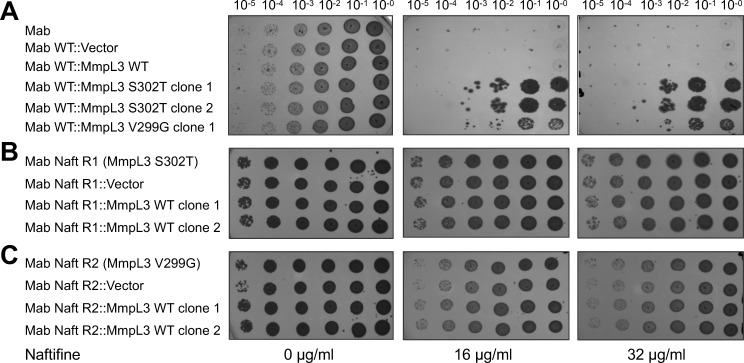
*MmpL3* mutations confer resistance to naftifine. Spot dilution assays demonstrate naftifine susceptibility across different *M. abscessus* strains and complementation constructs. Serial 10-fold dilutions (10^-5^ to 10^0^) of bacterial cultures were spotted onto 7H10-oleic albumin dextrose catalase agar plates containing no drug (0 μg/mL, left), 16 μg/mL naftifine (middle), or 32 μg/mL naftifine (right). (**A**) Wild-type *M. abscessus* ATCC 19977 (Mab) and strains transformed with vector control, wild-type MmpL3, or mutant MmpL3 alleles (S302T or V299G) show that expression of mutant MmpL3 variants confers naftifine resistance compared to wild-type controls. (**B**) Naftifine-resistant strain Naft R1 (harboring MmpL3 S302T mutation) and its derivatives transformed with vector control or wild-type MmpL3 clones demonstrate that complementation with wild-type MmpL3 does not restore naftifine sensitivity. (**C**) Naftifine-resistant strain Naft R2 (harboring MmpL3 V299G mutation) and its complemented derivatives show that wild-type MmpL3 expression cannot overcome the resistance conferred by the V299G mutation. Colony growth was assessed after 4 days of incubation, indicating that these MmpL3 mutations act dominantly to confer naftifine resistance.

Comparative genomic sequence analysis revealed single-nucleotide polymorphisms in the *MAB_4508* gene, which encodes the putative membrane transporter MmpL3 (Mycobacterial membrane protein Large 3). Specifically, the Naft R1 mutant harbored a Ser302Thr (S302T) substitution, while the Naft R2 mutant carried a Val299Gly (V299G) mutation. To verify MmpL3 as a potential target of naftifine, we cloned three alleles of the *MAB_4508* gene under the constitutive *hsp60* promoter for expression in *M. abscessus*: the wild-type allele and two mutant variants encoding the S302T and V299G substitutions. Expression of the wild-type MmpL3 did not affect naftifine susceptibility relative to the control strain harboring the empty vector (pMV261). In contrast, the expression of both mutant MmpL3 variants conferred significantly increased resistance to naftifine (Fig. 6A). Notably, reintroduction of the wild-type *MAB_4508* gene into the resistant Naft R1 and Naft R2 strains did not restore susceptibility to naftifine (Fig. 6B and C). This indicates that the mutant form of MmpL3 remains functionally competent despite the presence of naftifine, strongly supporting MmpL3 as the likely molecular target of the compound.

### Cross-resistance of naftifine-resistant *M. abscessus* to structurally distinct MmpL3 inhibitors

**Fig 7:**
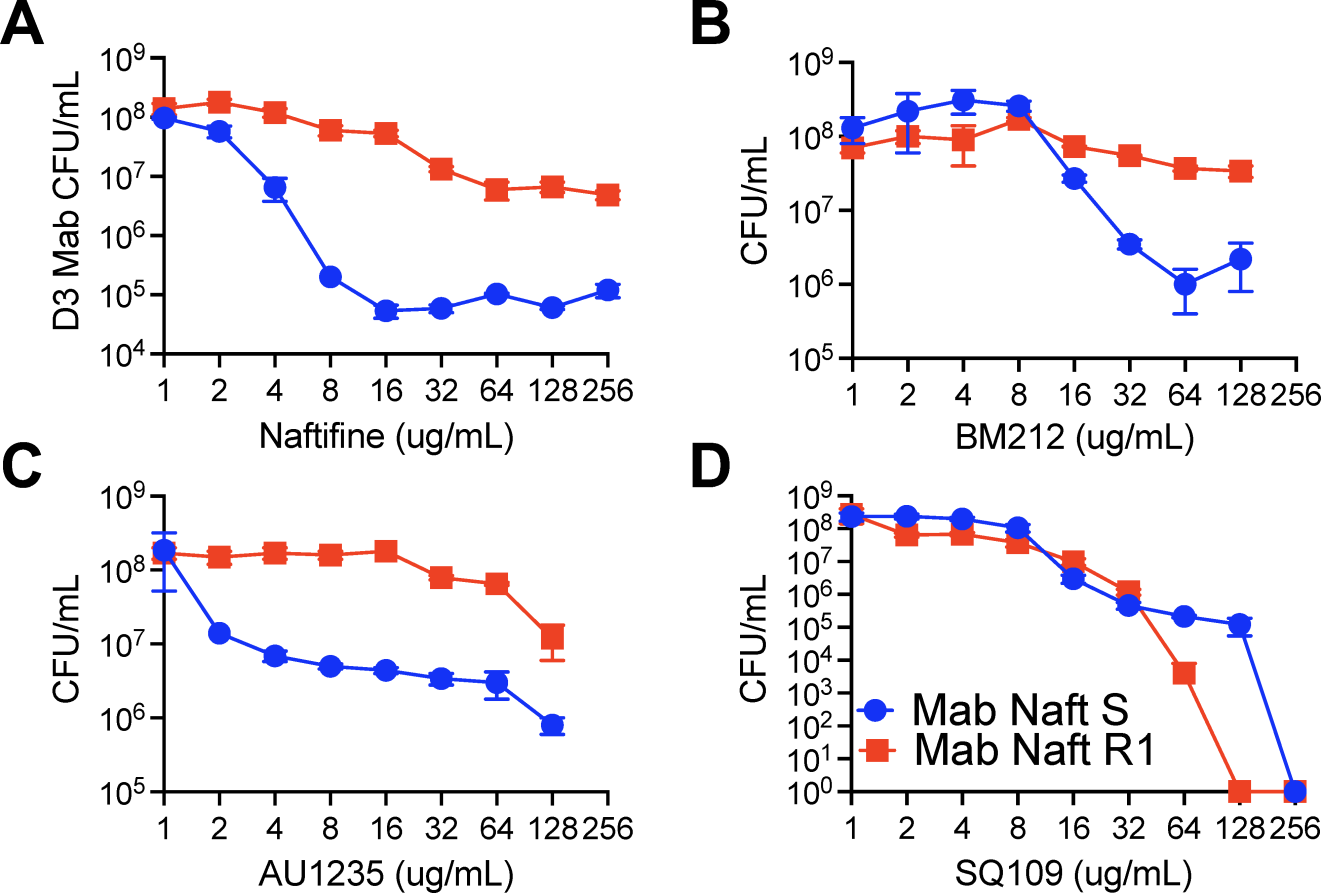
Cross-resistance of naftifine-resistant *M. abscessus* to structurally distinct MmpL3 inhibitors. Dose-response curves show CFU/mL of naftifine-sensitive (Naft S, blue circles) and naftifine-resistant (Naft R1, red squares) *M. abscessus* strains exposed to serial twofold dilutions (1–128 or 256 μg/mL) of four MmpL3-targeting compounds: naftifine (**A**), BM212 (**B**), AU1235 (**C**), and SQ109 (**D**). Bacterial viability was assessed by CFU enumeration after 3 days of drug treatment, with data points representing mean CFU/mL ± SEM plotted on a logarithmic scale.

To determine whether the naftifine-resistant Naft R1 strain exhibits cross-resistance to other MmpL3 inhibitors, we compared its drug sensitivity to the naftifine-sensitive (Naft S) strain using three well-characterized MmpL3-targeting compounds: BM212 (an indole-2-carboxamide derivative), AU1235 (a piperidine-based benzamide), and SQ109 (a 1,2-diamine ethambutol analog), across a range of concentrations (Fig. 7A through D).

As expected, Naft R1 displayed pronounced resistance to naftifine, showing higher survival than Naft S at all concentrations tested (Fig. 7A). A similar resistance profile was observed with both BM212 (Fig. 7B) and AU1235 (Fig. 7C), where Naft R1 maintained significantly greater viability than Naft S, particularly at mid to high drug concentrations. These findings indicate that the mutation conferring naftifine resistance in Naft R1 also reduces susceptibility to structurally distinct but mechanistically related MmpL3 inhibitors. In contrast, both Naft S and Naft R1 displayed comparable sensitivity to SQ109 (Fig. 7D), as reflected by overlapping dose-response curves across all tested concentrations. This lack of differential susceptibility suggests that SQ109 may bind to MmpL3 at a distinct site or inhibit its function through a mechanism that is not affected by the resistance-conferring mutation in Naft R1. Additionally, SQ109 is known to act on multiple cellular targets, including the disruption of membrane potential (44–47) and inhibition of menaquinone biosynthesis (45), which may contribute to its retained activity in the resistant strain. Collectively, these results support MmpL3 as the primary molecular target of naftifine and confirm that the Naft R1 resistance mutation broadly affects sensitivity to multiple MmpL3 inhibitors. However, the absence of cross-resistance to SQ109 highlights mechanistic divergence within the MmpL3 inhibitor class. These findings underscore the central role of MmpL3 in drug development against *M. abscessus* and point to challenges and opportunities in designing next-generation MmpL3 inhibitors.

### Naftifine activates autophagy in *M. abscessus-*infected macrophages

**Fig 8:**
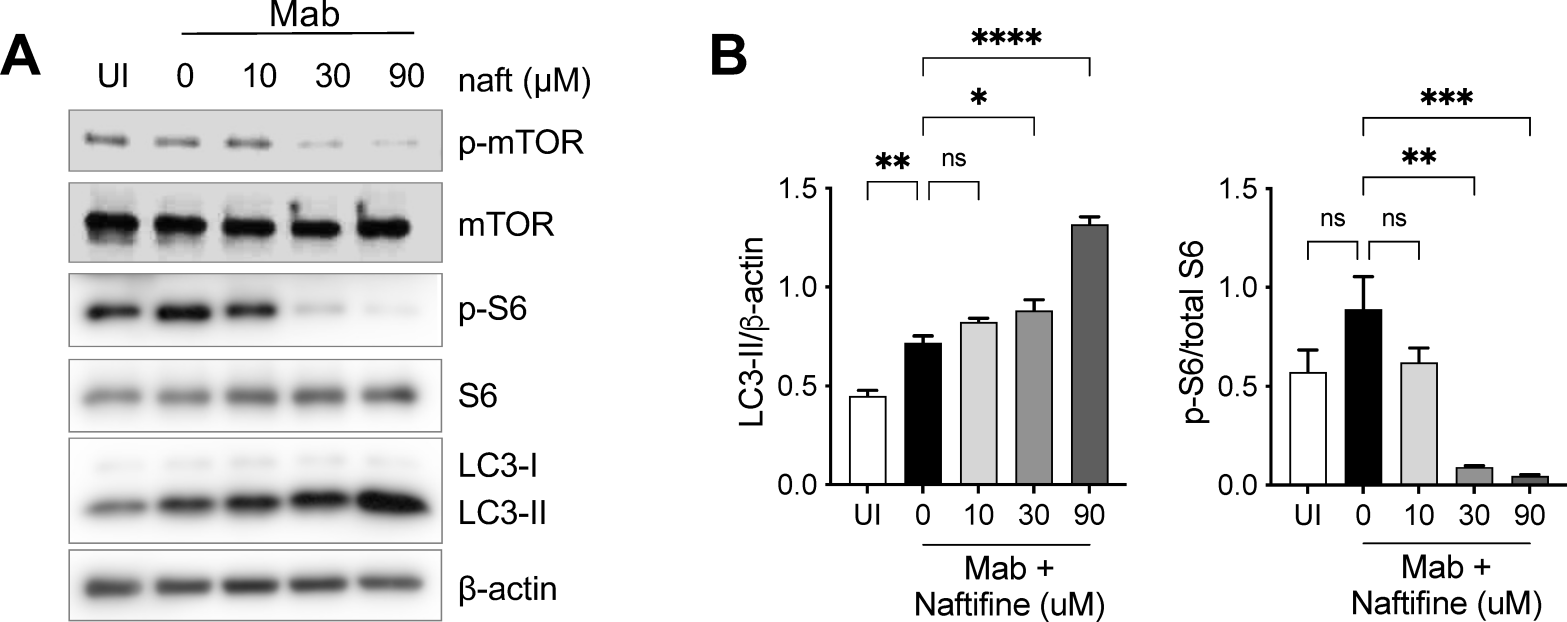
Naftifine promotes autophagy by inhibiting mTOR signaling in *M. abscessus*-infected macrophages. (**A**) Representative immunoblots showing levels of phosphorylated (p-mTOR), total mTOR, phosphorylated S6 ribosomal protein (P-S6), total S6, LC3-I and LC3-II, and β-actin (loading control) in THP-1-derived macrophages at 24 hours post-infection with *M. abscessus* and treatment with naftifine at indicated concentrations (0, 10, 30, or 90 μM). (**B**) Quantification of LC3-II levels normalized to β-actin and the ratio of p-S6 to total S6 from immunoblot data. Data represent mean ± SEM from multiple independent experiments. Statistical significance was determined by one-way ANOVA with *post hoc* analysis, comparing each treatment group to the vehicle DMSO control (0 μM naftifine). *P < 0.05, **P < 0.01, ***P < 0.005, and ****P < 0.001. UI, uninfected; ns, not significant.

Autophagy is a vital cellular process that degrades and recycles damaged organelles and intracellular pathogens, playing a crucial role in host defense against mycobacterial infection, including NTM. To explore whether autophagy contributes to naftifine’s antimycobacterial activity, we examined molecular markers associated with autophagic activation. Naftifine treatment significantly increased LC3-II levels, an established marker of autophagosome formation, in *M. abscessus*-infected THP-1 macrophages at 24 hours post-treatment (Fig. 8). LC3-II, the lipidated form of the microtubule-associated protein 1 light chain 3 (LC3), is widely recognized as a reliable indicator of autophagy induction (48). In parallel, naftifine markedly inhibited the mTOR signaling pathway, as evidenced by decreased phosphorylation of both mTOR and its downstream effector, ribosomal protein S6, which are key markers of pathway activation (49–51).

In uninfected THP-1 cells, naftifine also induced LC3-II accumulation in a dose-dependent manner (Fig. S1), further supporting its role in promoting autophagy. Additional treatment with bafilomycin A1 (BafA1), a V-ATPase inhibitor that blocks autophagosome-lysosome fusion, led to a further increase in LC3-II levels in naftifine-treated cells. This additive effect indicates that naftifine promotes autophagosome formation rather than inhibiting downstream autophagic flux. Together, these results suggest that naftifine activates host autophagy, which may contribute to its ability to control intracellular *M. abscessus*.

To investigate whether naftifine’s intracellular antimycobacterial activity is mediated through host autophagy, we compared its effects in wild-type RAW 264.7 macrophages and ATG16L1 knockdown (ATG16L1 KD) macrophages, which have impaired autophagic function (Fig. S2). We assessed intracellular bacterial burdens of both the naftifine-sensitive ATCC 19977^T^ strain (Naft S) and the naftifine-resistant strain Naftifine R1 (Naft R1). Cells were treated with increasing concentrations of naftifine (5, 10, and 20 µM) for days 1 and 2 post-infections.

**Fig 9:**
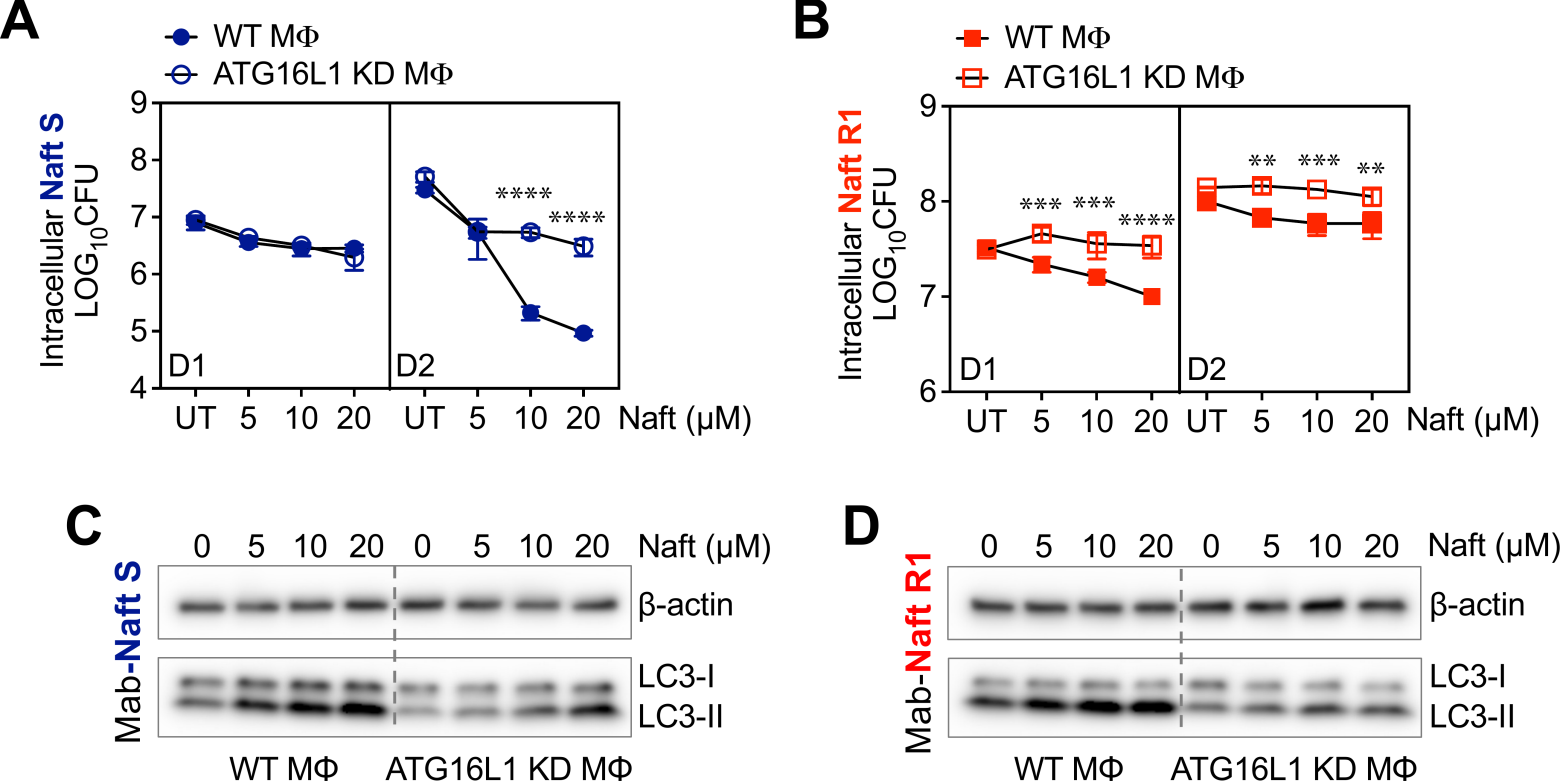
Autophagy contributes to the intracellular efficacy of naftifine against *M. abscessus*. Intracellular bacterial survival (log_10_CFU) of (**A**) naftifine-sensitive *M. abscessus* ATCC 19977^T^ S (Mab S, blue) strain and naftifine-resistant *M. abscessus* strain (Mab-Naft R1, red) in wild-type RAW 264.7 macrophages (filled circles) or (**B**) ATG16L1 knockdown macrophages (open circles) treated with naftifine at 0, 5, 10, and 20 µM. Bacterial loads were quantified on days 1 (D1) and 2 (D2) post-infection. (**C and D**) Representative immunoblots showing LC3-I/LC3-II expression and β-actin (loading control) in macrophage lysates at day 1 post-infection with (**C**) Mab-Naft S or (**D**) Mab-Naft R1, treated with indicated naftifine concentrations (μM). Data represent mean ± SEM. Statistical significance was determined by two-way ANOVA. **P < 0.01, *** *P* < 0.001, and ****P < 0.0001.

For the Naft S strain, naftifine reduced intracellular CFUs in both wild-type and ATG16L1 KD cells in a dose-dependent manner. However, the reduction was more pronounced in wild-type macrophages, particularly on day 2, indicating that autophagy enhances but is not strictly required for controlling the sensitive strain (Fig. 9A). In contrast, for the Naft R1 strain, naftifine significantly reduced bacterial burden in wild-type cells at both time points, while no reduction was observed in ATG16L1 KD cells (Fig. 9B). Moreover, bacterial loads in knockdown cells remained consistently higher across all naftifine concentrations and time points, suggesting that autophagy is essential for naftifine’s activity against this resistant strain. To confirm autophagy induction, we evaluated LC3-II levels by immunoblotting. Naftifine treatment increased LC3-II in wild-type RAW cells in a dose-dependent manner for both strains (Fig. 9C and D), consistent with autophagosome accumulation. In ATG16L1 KD cells, LC3-II levels were markedly reduced regardless of treatment, confirming disruption of the autophagy pathway.

Together, these data demonstrate that naftifine activates host autophagy, and this pathway is necessary for controlling intracellular *M. abscessus*, particularly in strains with reduced drug sensitivity. While naftifine exerts both direct and host-mediated antimicrobial effects, autophagy appears to play a critical role in its intracellular activity.

### Naftifine interacts with other antibiotics

**Fig 10:**
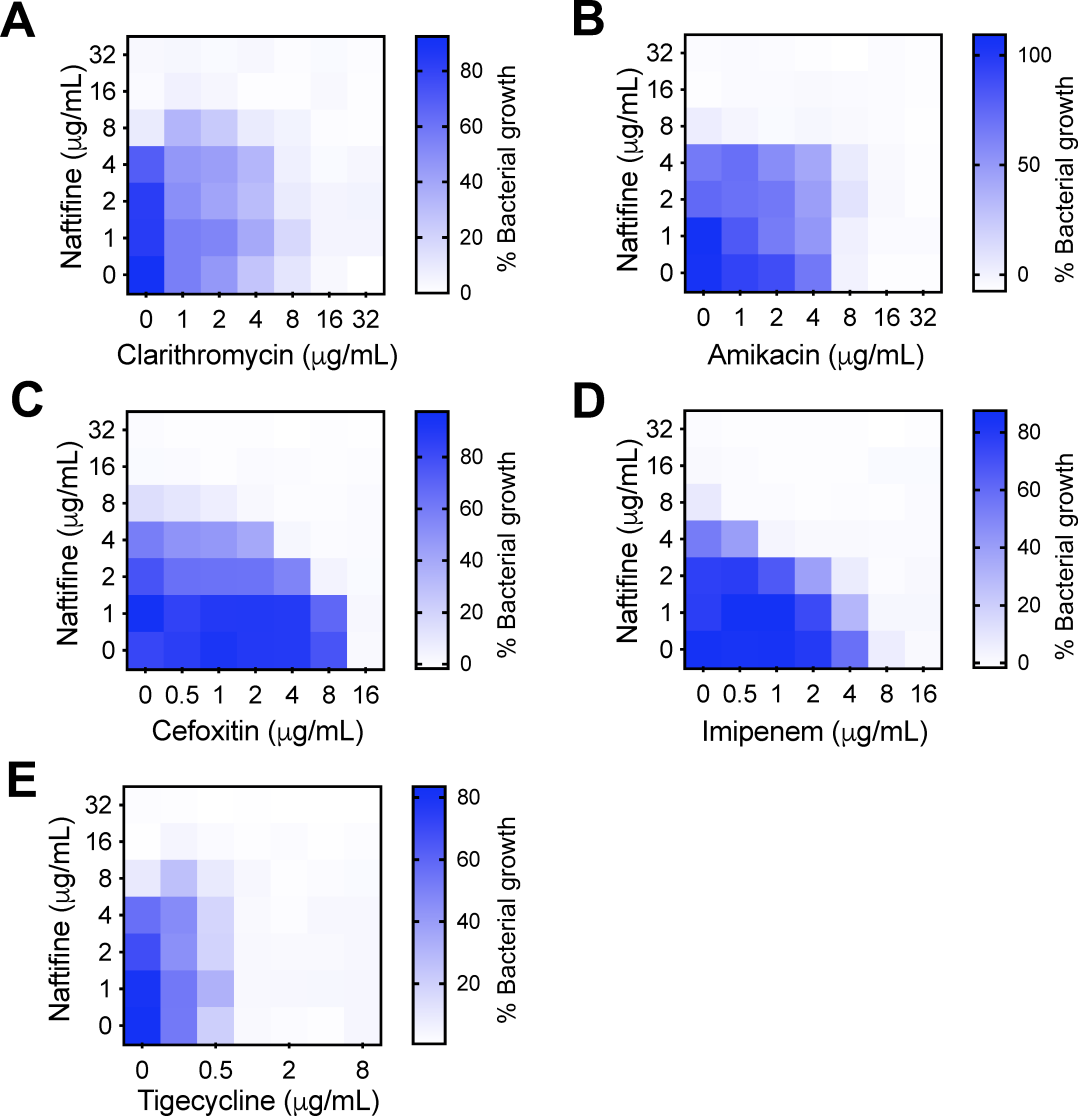
Synergistic interactions between naftifine and β-lactam antibiotics against *M. abscessus*. Checkerboard assays were performed to evaluate drug interactions between naftifine (*y*-axis) and various antibiotics (*x*-axis) against *M. abscessus* ATCC 19977^T^ smooth variant. Heat maps display the percentage of bacterial growth, with darker blue indicating greater growth. Twofold serial dilutions of each drug were tested in combination, and growth inhibition was assessed after incubation. Naftifine showed indifferent interactions with clarithromycin (**A**) and tigecycline (**E**), additive/partial synergistic effects with amikacin (**B**), but demonstrated synergistic effects when combined with the β-lactam antibiotics cefoxitin (**C**) and imipenem (**D**), particularly at sub-inhibitory concentrations. Synergy was defined as FICI ≤ 0.5, where the combined effect significantly exceeded the sum of individual drug activities. Data were analyzed and plotted using GraphPad Prism version 10.

Since multidrug combination therapy is crucial for effectively treating *M. abscessus* infections, we conducted a checkerboard assay to evaluate the interactions between naftifine and several clinically used antibiotics. Combinations of naftifine with clarithromycin, amikacin, or tigecycline demonstrated indifferent interactions (Fig. 10A, B, and E). However, naftifine exhibited synergistic effects when combined with the β-lactams cefoxitin and imipenem. The calculated fractional inhibitory concentration index (FICI) values were 0.5 for cefoxitin and 0.31 for imipenem, indicating clear synergy (FICI ≤ 0.5) (Fig. 10C and D; Table 2). These results suggest that naftifine does not interfere with the activity of key antibiotics used to treat *M. abscessus* and may improve the efficacy of β-lactam-based therapies when combined.

**TABLE 2:** Interaction of naftifine with other antibiotics against *M. abscessus* ATCC 19977^T^

Antibiotic	Individual treatment MIC (μg/mL)		Combination treatment MIC (μg/mL)			
	Drug	Naftifine	Drug	Naftifine	FICI	Indication
Clarithromycin	16	16	8	16	1.5	Indifference
Amikacin	8	16	1	8	0.625	Indifference
Cefoxitin	16	16	4	4	0.5	Synergism
Imipenem	16	16	1	4	0.31	Synergism
Tigecycline	1	16	0.5	16	1.5	Indifference

## DISCUSSION

Through a comprehensive screen of FDA-approved drugs, we identified naftifine, a topical antifungal agent, as a potent inhibitor of *M. abscessus* with both direct antibacterial and host-directed immunomodulatory activities (Fig. 1). This dual-action mechanism is critical given *M. abscessus’* intrinsic and acquired resistance to multiple antibiotics and the limited therapeutic success of current treatment regimens.

Our results show that naftifine has potent efficacy against the reference isolate ATCC 19977^T^ and a broad group of *M. abscessus* clinical isolates that harbor mutations conferring resistance to widely used drugs such as clarithromycin and amikacin (Table 1). In fact, such multidrug-resistant strains represent one of the most challenging types of infections in hospitals, with a failure rate of over 50%. In particular, naftifine’s efficacy in reducing intracellular bacteria in multidrug-resistant strains at concentrations as low as 3–10 μM (Fig. 2) identifies it as a promising therapeutic candidate against such infections.

One of the most marked findings of our study is that naftifine’s activity is higher under intracellular conditions than in axenic culture (Fig. 2 and 4). This increased intracellular activity appears to be, at least in part, related to naftifine’s ability to induce autophagy in infected macrophages. We demonstrated that naftifine treatment significantly increases LC3-II levels and suppresses mTOR signaling, key markers of autophagy induction (Fig. 8). The importance of this mechanism is underscored by experiments with ATG16L1 knockdown macrophages, which showed reduced efficacy of naftifine against intracellular bacteria, particularly the resistant strain (Fig. 9).

This autophagy-mediated mechanism is particularly relevant for *M. abscessus* infections, as the bacterium has evolved strategies to manipulate host autophagy for its survival. Previous studies have shown that azithromycin, a macrolide antibiotic included in current *M. abscessus* treatment regimens, paradoxically impairs autophagosome clearance, potentially promoting bacterial persistence (52). Conversely, compounds that enhance autophagy, such as the resveratrol analog V46, improve *M. abscessus* clearance both in macrophages and *in vivo* by inhibiting AKT/mTOR activation (53). Our results position naftifine as a unique therapeutic that simultaneously targets the pathogen directly while enhancing the host’s natural defense mechanisms (54, 55).

Beyond autophagy activation, naftifine treatment significantly improved macrophage survival during infection, reducing cell death from 59% to less than 20% even at the highest concentration tested (Fig. 3). This cytoprotective effect may limit bacterial dissemination, as *M. abscessus* can exploit macrophage necrosis to escape cellular containment and establish new foci of infection.

Our mechanistic study provides compelling evidence that MmpL3 (Mycobacterial membrane protein Large 3), encoded by *MAB_4508*, is a direct molecular target of naftifine (56). The isolation of spontaneous naftifine-resistant mutants harboring specific point mutations (S302T and V299G) in transmembrane helix V of MmpL3, combined with complementation studies demonstrating that expression of these mutant alleles confers high-level resistance, strongly implicates this transporter as naftifine’s target (Fig. 6). MmpL3 is an essential component of the mycobacterial cell wall biosynthesis machinery, responsible for translocating trehalose monomycolate (TMM) from the cytoplasm to the periplasmic space, where it is converted to trehalose dimycolate and integrated into the outer leaflet of the membrane. Mycolic acids from TMM also attach to arabinogalactan to form the inner leaflet of the membrane (56).

Of the 13 *MmpL* genes in *M. tuberculosis*, only MmpL3 is essential in strain H37Rv, and its depletion is lethal both *in vitro* and *in vivo* (57, 58). Multiple structurally diverse chemical classes have been identified as MmpL3 inhibitors, including diamines (e.g., SQ109), indole-2-carboxamides (e.g., ICA38), adamantyl ureas (AU1235), pyrroles (BM212), piperidinols (PIPD1), and benzimidazoles (e.g., C215) (59). Mycolic acid transport via MmpL3 is a conserved process in all *Mycobacterium* species. Inhibitors such as PIPD1, indole-2-carboxamides, and analogs have potent activity against *M. tuberculosis* and *M. abscessus* by inhibiting TMM secretion and cell wall integrity (10, 13, 60, 61).

To better understand the molecular basis of species-specific differences in drug susceptibility, Li et al. (61) conducted comparative studies using *M. smegmatis* recombinant strains expressing MmpL3 orthologs from different mycobacterial species. When MmpL3 from *M. tuberculosis*, *M. smegmatis*, and *M. abscessus* were expressed from identical promoters in the same *M. smegmatis* background strain (eliminating variables such as drug uptake, efflux, and modification), the MIC values of various MmpL3 inhibitors against these recombinant strains closely reflected their activity against the respective parent species. This elegant study showed that it is the structural differences among MmpL3 orthologs that define a pattern of susceptibilities, rather than interspecies differences in drug permeability or efflux pumps. Consistent with these findings, naftifine shows substantially higher activity against *M. abscessus* (MIC 16 μg/mL) compared to *M. tuberculosis* (MIC 64 μg/mL), reflecting the approximately 56% amino acid sequence identity between these orthologs (62). Structural analysis of existing crystalline structures and modeling studies have provided insight that MmpL3 is a periplasmic pore-containing protein with a transmembrane region consisting of 12 helices, in which 2 pairs of conserved Asp-Tyr residues (Asp256-Tyr646 and Asp645-Tyr257) are crucial for proton transfer. This suggests that divergent sequences of MmpL3 proteins may occur within transmembrane domains that overlap with ligand-binding domains, thereby contributing to differences in ligand-binding affinity among inhibitors of Mycobacteria (63–65).

Naftifine-resistant *M. abscessus* mutants were found to harbor S302T or V299G point mutations in transmembrane α helix V, a component of the binding pocket shared by SQ109, AU1235, ICA38, and rimonabant (63). The two residues S302 and V299 are among 18 that form a large binding cavity for benzimidazole analogs EJMCh-6 and BMC-2i (66). Naftifine-resistant *M. abscessus* Naft R1 strain is also resistant to other MmpL3 inhibitors, such as AU1235 and BM212 (Fig. 7). Interestingly, the resistant strain retained sensitivity to SQ109, suggesting that this compound may bind to a distinct site or employ an alternative mechanism of MmpL3 inhibition. Failure of wild-type MmpL3 to reverse naftifine resistance suggests that mutations have introduced a dominant-negative effect, likely altering protein conformation to prevent drug binding while preserving essential transport function. This observation has important implications for designing a combination therapy or a strategy to prevent the emergence of resistance.

An additional advantage of naftifine is its favorable interaction profile with existing anti-*M*. *abscessus* antibiotics. While showing indifferent interactions with clarithromycin, amikacin, and tigecycline, naftifine exhibited clear synergistic activity with β-lactam antibiotics cefoxitin and imipenem, key components of clinical regimens for *M. abscessus,* with FICI values of 0.5 and 0.31, respectively (Fig. 10; Table 2). This synergistic effect is mechanistically logical, as β-lactams inhibit peptidoglycan synthesis, while naftifine prevents mycolic acid uptake, thus offering a complementary mode of cell wall disruption. This synergistic effect may offer a means to reduce drug doses, thereby mitigating toxicities associated with the current 12–18 months of multidrug therapy in *M. abscessus* infections.

In our immunosuppressed mouse model, naftifine treatment (50 mg/kg of body weight daily for 14 days) resulted in significant reductions in bacterial burden in both lungs and spleens, demonstrating clear *in vivo* antimicrobial activity comparable to the reference antibiotic cefoxitin (Fig. 5). The histopathological studies revealed that naftifine-treated mice exhibited a marked decrease in inflammatory pathologies as well as a well-preserved architecture of the lung compared to those that received no treatment, thus supporting that the compound’s action is a combination of antimicrobial and anti-inflammatory activities. Although the observed bacterial reductions were modest, they provide proof of concept for the therapeutic efficacy of naftifine. Naftifine’s hydrophobicity might hinder its systemic absorption, but this could be addressed through innovative formulation or structure-activity relationship studies. Considering that naftifine is known to be non-toxic when applied topically as an antifungal, its new therapeutic use in an inhaled form could target *M. abscessus* in the respiratory tract while avoiding systemic absorption.

There are several important questions that remain to be answered in future studies. First, a detailed analysis of naftifine’s molecular mechanism of action against MmpL3 is needed. This is because insight into naftifine’s action against TMM translocation, protein stability, or proton motive force could improve analog designs (13, 65, 66). Second, a continued challenge is the development of resistance, as evidenced by the identification of highly resistant mutants in this study. Future studies will explore combination designs that maximize resistance avoidance and test whether naftifine’s ability to induce autophagy serves as a rescue factor against target-based resistance. Third, a complete characterization of naftifine’s immune-modulatory effects will be beneficial. The global impacts of naftifine on an organism’s immune system will need to be assessed using techniques such as transcriptomic and phosphoproteomic analyses of downstream signaling pathways following mTOR suppression. Finally, structure-activity relationship analysis will need to aim to optimize pharmacokinetics in naftifine while preserving its two-part mode of action.

* *In summary, naftifine represents a novel dual-acting therapeutic that simultaneously inhibits bacterial growth through MmpL3 targeting and enhances host immune responses through autophagy activation. While MmpL3 inhibitors (67) and autophagy inducers (68) have individually shown antimicrobial promise, naftifine is, to our knowledge, the first compound to employ both mechanisms simultaneously. Its synergistic interactions with β-lactam antibiotics and its efficacy against models of cellular and animal infections make naftifine a compound of interest for use in adjunct therapy against *M. abscessus*, a bacterium known for its high inherent and acquired resistance to antibiotics. This study identifies an emerging trend in antimicrobial therapy that combines pathogen-specific and host defense mechanisms, potentially offering therapeutic advantage in infections caused by these pathogens.

## MATERIALS AND METHODS

### Bacterial strains and culture media

All *M. abscessus* strains were cultured in Middlebrook 7H9 (BD, Franklin Lakes, NJ, USA) liquid medium supplemented with 0.2% glycerol, 0.05% tyloxapol, and 10% oleic albumin dextrose catalase (OADC) at 37°C with shaking at 140 rpm. The reference strain *M. abscessus* ATCC 19977^T^ was obtained from the American Type Culture Collection (ATCC, Manassas, VA, USA). Smooth and rough morphotypes were isolated by streaking the stock onto Middlebrook 7H10 plates supplemented with 10% OADC and glycerol, followed by selection of individual colonies. Glycerol frozen stocks of each variant were prepared in PBS containing 15% glycerol and stored at −80°C. All clinical isolates of *M. abscessus* were kindly provided by Prof. Barbara Brown-Elliott from The University of Texas at Tyler.

For drug screening, a fluorescent reporter strain of *M. abscessus* ATCC 19977^T^ was generated by transforming the bacterium with a modified pTEC27-tdTomato containing an apramycin selection marker. The original pTEC27-tdTomato, containing a hygromycin selection marker, was generously provided by Dr. David Tobin (Duke University, Durham, NC, USA). Electroporation was performed using a Gene Pulser system (Bio-Rad, Hercules, CA, USA) under the following conditions: 2.5 kV, 25 µF, and 1,000 Ω. Transformed bacteria were selected on Middlebrook 7H10 agar supplemented with 10% OADC, glycerol, and apramycin (100 µg/mL). A single colony was picked and propagated in 7H9 broth with OADC and apramycin. Cultures grown to the logarithmic phase were aliquoted and stored at –80°C for further use.

### Chemicals

The Screen-well FDA-approved drug library V2 (Enzo Life Sciences, Farmingdale, NY, USA) comprises 786 drugs, each supplied at a concentration of 10 mM in DMSO. Phorbol 12-myristate 13-acetate, naftifine hydrochloride, bedaquiline, imipenem, cefoxitin, clarithromycin, tigecycline, amikacin, and dexamethasone were all purchased from Sigma-Aldrich (St. Louis, MO, USA). Bafilomycin A1 was purchased from Cell Signaling Technology (Danvers, MA, USA). The MmpL3 inhibitors AU1235, SQ109, and BM212 were purchased from MedChemExpress (Monmouth Junction, NJ, USA).

### Cell culture

THP-1 monocytes were cultured in RPMI 1640 medium supplemented with 10% heat-inactivated fetal bovine serum, 1% nonessential amino acids, 1 mM sodium pyruvate, and 10 mM HEPES at 37°C with 5% CO_2_. To prepare adherent macrophages for infection, monocytes were differentiated by treating them with 40 ng/mL phorbol 12-myristate 13-acetate in complete RPMI for 24 hours. After differentiation, the adherent cells were washed in complete RPMI medium and allowed to rest for an additional 24 hours before infection. ATG16L1 (autophagy-related 16 like 1) knockdown and corresponding control RAW264.7 cells were previously described (69). RAW264.7 cells were cultured in high-glucose Dulbecco’s modified Eagle medium supplemented with 1% nonessential amino acids, 10% heat-inactivated fetal bovine serum, and 50 μM β-mercaptoethanol. Cells were maintained at 37°C with 5% CO_2_.

### High-content imaging for drug screening

The Screen-Well FDA-approved drug library V2 was used for high-throughput screening. Human THP-1 monocytes were infected with *M. abscessus*-tdTomato at a multiplicity of infection (MOI) of 5 in suspension at 37°C with shaking at 130 rpm for 4 hours, concurrently undergoing differentiation with 40 ng/mL PMA in batch culture. After infection, cells were pelleted down and washed three times with antibiotic-free RPMI 1640 medium (Sigma-Aldrich), then seeded into 96-well black, clear flat-bottom plates (Corning, Corning, NY, USA) that were pre-loaded with drugs to a final concentration of 10 μM per well. Cells were incubated for 48 hours at 37°C with 5% CO_2_. Following incubation, the medium was removed, and cells were fixed and stained with Hoechst 33342. Imaging was performed using PerkinElmer Opera Phenix High Content Screening system (10× objective), capturing five fields per well across the Hoechst 33342 (nuclear) and tdTomato fluorescence channels. Image analysis was conducted using Harmony software, which quantified total cell numbers and the percentage of infected cells based on red fluorescence.

The assay was calibrated using 0.1% DMSO as a drug vehicle, negative control (representing 100% bacterial growth), and bedaquiline at 1.5 μM as a positive control (representing 100% bacterial inhibition). Cell viability was calculated as the percentage of surviving cells compared to negative control wells. Percent inhibition was calculated using the formula: % inhibition = 100 × ([signal_DMSO_ − signal_sample_]/[signal_DMSO_ − signal_bedaquiline_]). The robustness of the assay was evaluated using the *Z*′ factor, calculated as *Z*′ = 1 – (3[SD_bedaquiline_ + SD_DMSO_]/[M_bedaquiline_ – *M*_DMSO_]), where SD represents standard deviation and *M* represents the average signal intensity for each control group.

### Evaluation of intracellular bacterial growth

THP-1 cells were infected with *M. abscessus* ATCC 19977^T^ smooth or rough variants, or with clinical isolates, at an MOI of 5 in the presence of 40 ng/mL PMA. Infections were carried out for 4 hours at 37°C with shaking. Following infection, cells were spun down and washed three times with complete RPMI medium without antibiotics, then seeded into 96-well plates pre-loaded with various concentrations of naftifine or clarithromycin. At 24, 48, and 72 hours post-infection, cells were washed thrice with PBS and lysed with RIPA buffer (Sigma-Aldrich). The cell lysates were serially diluted in PBS and plated on Middlebrook 7H10 plates supplemented with 10% OADC and glycerol. Plates were incubated at 37°C for 5–7 days, after which CFUs were counted to quantify intracellular bacterial burden.

### Apoptosis and necrosis detection analysis

A GFP-certified apoptosis/necrosis detection kit (Enzo Life Sciences) was used to assess cell death. An Annexin V-EnzoGold was conjugated with enhanced Cyanine 3 to detect externalized phosphatidylserine, which is a marker of early apoptosis. The necrosis detection reagent, 7-AAD, binds to the double-stranded DNA of dead cells or cells with compromised membranes, facilitating the detection of late apoptosis/necrosis. Briefly, following treatment with or without *M. abscessus* infection, all cells were harvested using trypsinization and washed. Cells were stained with apoptosis and necrosis detection reagents in 1× binding buffer at room temperature in the dark for 15 minutes. Samples were then analyzed using a BD Accuri C6 plus flow cytometer. Non-viable cells were defined as those positive for Annexin V, regardless of 7-AAD staining status. This includes both Annexin V^+^/7-AAD⁻ (early apoptotic) and Annexin V^+^/7-AAD^+^ (late apoptotic or necrotic) populations. Viable cells were negative for both Annexin V and 7-AAD.

### Immunoblotting

After infection or drug treatments, cells were washed once with PBS and lysed in 1× RIPA buffer. Protein lysates were clarified by centrifugation, and the resulting supernatants were mixed with 4× Laemmli buffer (Bio-Rad) and boiled for 5 minutes to denature the proteins. The denatured proteins were resolved on 12% SDS-PAGE gels and then transferred to 0.2 μm polyvinylidene difluoride membranes using the Bio-Rad Transblot Turbo system. After transfer, these membranes were blocked in OneBlock Western-CL blocking buffer (Genesee Scientific, Morrisville, NC, USA) for 1 hour at room temperature, followed by overnight incubation at 4°C with primary antibodies (Cell Signaling Technology, Danvers, MA, USA), including LC3B (Cat#2775), phospho-S6 Ser 235/236 (Cat#4857), S6 (Cat#2217), p-mTOR S2448 (Cat#2971), mTOR (Cat#2983), and β-actin (Cat#4970). After washing, membranes were incubated with horseradish peroxidase-conjugated anti-rabbit IgG secondary antibody for 1 hour at room temperature. Clarity ECL substrate (Bio-Rad) was used to visualize the proteins of interest, and images were captured with Amersham Imager 680 (GE Healthcare Life Sciences, Marlborough, MA, USA). Densitometric analysis was conducted using ImageJ software (NIH) to quantify band intensities.

### Antimicrobial susceptibility testing

The susceptibility of *M. abscessus* strains to naftifine and other MmpL3 inhibitors (AU1235, BM212, and SQ109) was determined using the microbroth dilution method. Log-phase bacteria grown in 7H9 broth supplemented with 10% OADC were diluted to 5 × 10^5^ CFU/mL and seeded into 96-well plates. Each compound was added to achieve final concentrations ranging from 128 to 0.25 μg/mL in twofold serial dilutions. Plates were incubated at 37°C for 3–4 days. The MIC was defined as the lowest drug concentration at which no visible bacterial growth was observed.

### Time-kill assay

Log-phase cultures of *M. abscessus* were diluted to an OD_600_ of 0.005 and cultured in 7H9 broth supplemented with 10% OADC, containing varying concentrations of naftifine. Cultures were incubated at 37°C, and at the indicated time points, aliquots were serially diluted in PBS and plated onto 7H10 agar plates supplemented with 10% OADC. Plates were incubated at 37°C for 5–7 days, and CFUs were counted to assess bacterial viability.

### Animal experiments

Female BALB/c mice (6–8-week-old, Jackson Laboratories, Bar Harbor, ME, USA) were administered 5 mg dexamethasone/kg of body weight subcutaneously once daily, beginning 1 week before intranasal infection and continuing throughout the study to induce immunosuppression. *M. abscessus* ATCC 19977^T^ R, grown to the logarithmic phase, was used for infection and delivered intranasally. Five mice were sacrificed 4 hours post-infection to confirm pulmonary infection, and lung homogenates were evaluated for bacterial load.

Naftifine (50 mg/kg of body weight) or PBS vehicle control was initiated 3 days post-infection and administered once daily via intraperitoneal injection. Cefoxitin (200 mg/kg of body weight, positive-control antibiotic) was administered once daily by subcutaneous injection. After 2 weeks of treatment, mice were sacrificed, and lungs and spleens were collected and homogenized. Undiluted and 10-fold serial dilutions of tissue homogenates were plated onto 7H10 agar supplemented with OADC. Plates were incubated at 37°C for 5 days, after which CFUs were counted to determine bacterial burden.

For histological analysis, lung tissue samples were fixed in 10% neutral-buffered formalin for 48 hours before being embedded in paraffin blocks. To ensure unbiased histopathological evaluation, standardized lung sections were prepared and stained using hematoxylin and eosin. These sections were then examined for infection-associated lesions and any evidence of fibrosis.

### Generation of spontaneous resistant mutants and whole-genome sequencing

Spontaneous naftifine-resistant mutants were selected by plating *M. abscessus* on 7H10 agar supplemented with 10% OADC and increasing concentrations of naftifine, ranging from 2× to 32× MIC. Resistant mutants were isolated, and genomic DNA was extracted for whole-genome sequencing, which was performed by next-generation sequencing with MiniSeq at the UTMB core facility.

### Constructs and recombinant *M. abscessus* strains

The full-length *MAB_4508* gene from *M. abscessus* ATCC 19977^T^ S (wild type) as well as from the naftifine-resistant Naft R1 and Naft R2 mutants was amplified by PCR and cloned in-frame to the mycobacterial expression vector pMV261. The resulting overexpression constructs were verified by DNA sequencing and electroporated into *M. abscessus* wild-type, Naft R1, or Naft R2 strains. Transformants were selected on 7H10 agar containing kanamycin (100 µg/mL). Single colonies were selected and expanded in 7H9 broth supplemented with OADC and kanamycin.

### Naftifine efficacy against intracellular *M. abscessus* in autophagy-deficient cells

ATG16L1 knockdown (ATG16L1 KD) and corresponding control RAW264.7 wild-type cells were plated into wells of 24-well plates 1 day prior to infection with *M. abscessus* ATCC 19977^T^ S and naftifine-resistant Naft R1 strains. Cells were then infected with *M. abscessus* for 3 hours at 37°C under 5% CO_2_ with an MOI of 2. Following infection, cells were washed three times with PBS and incubated with 0, 5, 10, or 20 μM naftifine for 1 or 2 days. After incubation, the cells were washed three times with PBS and lysed with RIPA buffer. The resulting samples were serially diluted with PBS and plated on 7H10 agar plates for CFU counts, allowing for the quantification of the intracellular bacterial burden. Additionally, lysates were subjected to immunoblotting to measure LC3-II levels within the cells.

### Checkerboard assay

Drug combination studies were performed using the checkerboard assay as previously described (70). In 96-well plates, twofold serial dilutions of naftifine were prepared along the ordinate (vertical axis) in Middlebrook 7H9 broth supplemented with 10% OADC, while twofold serial dilutions of the second antibiotic were prepared along the abscissa (horizontal axis). Log-phase *M. abscessus* cultures were harvested, adjusted to 5 × 10^5^ CFU/mL in the same medium, and 100 μL of bacterial suspension was added to each well. After incubation at 37°C for 3 days, the OD_600_ of each well was measured with a Victor Nivo multimode plate reader (PerkinElmer, Waltham, MA, USA). The MIC was defined as the inhibition of 90% of bacterial growth. The FICI was calculated using the formula: FICI = (MIC of drug A in combination/MIC of drug A alone) + (MIC of drug B in combination/MIC of drug B alone). Interpretation was as follows: FICI ≤ 0.5 indicated synergy; 0.5 <FICI ≤ 4 indicated indifference; and FICI > 4 indicated antagonism.

### Statistical analysis

All experiments were repeated two to three times with triplicate samples. All statistical analyses were performed using GraphPad Prism version 7. For comparisons involving more than two groups, statistical significance was evaluated using either the non-parametric Kruskal–Wallis test or the parametric one-way or two-way ANOVA, depending on data distribution and variance homogeneity. When significant differences were detected, *post hoc* tests were used to compare individual group pairs. A *P*-value less than 0.05 was considered statistically significant.

## SUPPLEMENTAL MATERIAL

Supplemental figures aac.01105-25-s0001.pdf Fig. S1 and S2.

ASM does not own the copyrights to Supplemental Material that may be linked to, or accessed through, an article. The authors have granted ASM a non-exclusive, world-wide license to publish the Supplemental Material files. Please contact the corresponding author directly for reuse.
