# Supplementary material for: Protective mechanism of action of the antifungal drug naftifine against Mycobacterium abscessus infection
Source: Antimicrob Agents Chemother. 2026 Jan 14;70(2):e01105-25. doi: 10.1128/aac.01105-25 (PMC12888883; doi:10.1128/aac.01105-25)
Supplement: Supplemental figures — Fig. S1 and S2. [file aac.01105-25-s0001.pdf]

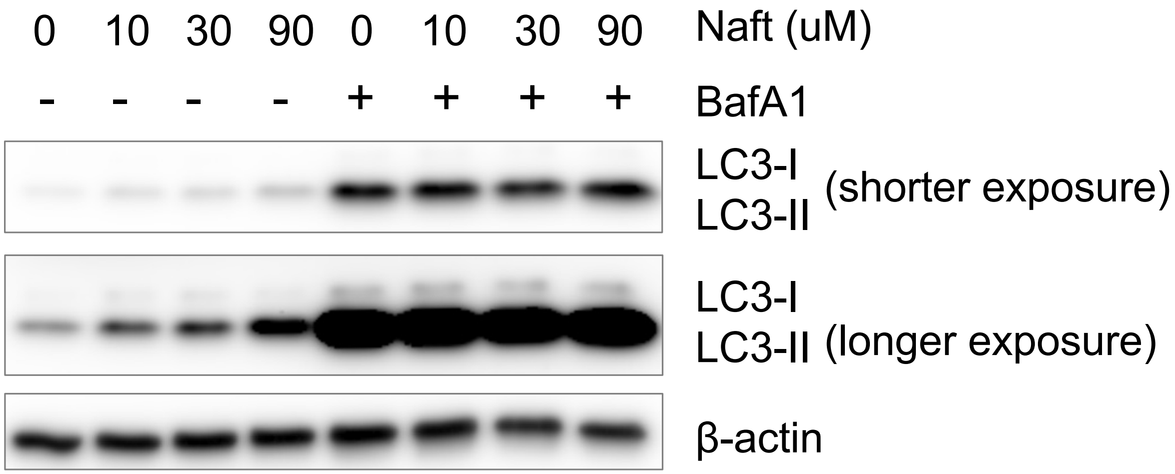

Supplemental Figure 1. Naftifine induces autophagy in uninfected macrophages. Representative immunoblots showing LC3-I and LC3-II levels in THP-1 derived macrophages 24 hours post-treatment with naftifine at concentrations of 10, 30, or 90 µM in the absence or presence of the V-ATPase inhibitor bafilomycin A1 (BafA1). LC3-II accumulation in the presence of bafilomycin A1 indicates autophagosome formation, while β-actin serves as a loading control. Two different exposure times are shown to demonstrate the dose-dependent increase in LC3-II levels. Naft, Naftifine.

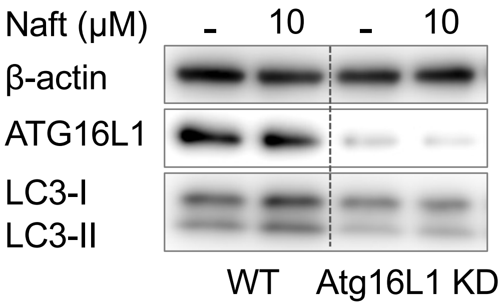

Supplemental Figure 2. Autophagy contributes to the intracellular efficacy of naftifine against *M. abscessus.* Representative immunoblots showing the expression levels of ATG16L1 and LC3-I/LC3-II in RAW 264.7 wildtype (WT) and ATG16L1 knockdown (Atg16L1 KD) cells treated with or without 10 μM naftifine. β-actin serves as a loading control. The reduced ATG16L1 expression in knockdown cells confirms successful autophagy pathway disruption, while LC3 conversion demonstrates naftifine-induced autophagy in wildtype but not in ATG16L1-deficient cells. Naft, Naftifine.
